# Extensor digitorum brevis flap on the treatment of lower limb injuries

**DOI:** 10.1590/1413-78522014220200862

**Published:** 2014

**Authors:** Luciano Ruiz Torres, Priscilla Messias Paganelli, Renan Pires Negrão dos Santos, Walter Hamilton de Castro Targa, Tulio Diniz Fernandes, Rames Mattar

**Affiliations:** USP, FM, Hospital das Clínicas, São Paulo, SP, Brazil, Institute of Orthopedics and Traumatology, Hospital das Clínicas, FMUSP, São Paulo, SP, Brazil

**Keywords:** Wounds and injuries, Surgical flaps, Osteomyelitis

## Abstract

**OBJECTIVE:**

To describe our pioneer national experience with 11 patients with soft tissue defects in the distal 1/3 of the leg, ankle and forefoot treated with extensor digitorum brevis muscle flap (EDB).

**METHODS:**

Between November 2009 and July 2012 11 patients were operated with the flap technique. We operated nine men and two women aged between 10 and 66 years. The surgical indications were acute trauma in four patients and post-traumatic osteomyelitis in seven patients. The small defects were covered ranging from 3x3 to 6x3 cm. The patch was applied with proximal stalk in most cases.

**RESULTS:**

Complete healing and infectious cure were obtained in all cases, despite one loss.

**CONCLUSION:**

The EDB flap is a feasible and safe technique to repair foot, ankle and distal leg losses. Suffering, dehiscence and delayed healing of the EDB end flap donor area may, however, occur. L-shaped incisions should be avoided for muscle lifting. ***Level of Evidence IV, Case series.***

## INTRODUCTION

Wounds in the foot and ankle determine the most complex area of coverage for reconstruction of lower limbs. The presence of terminal vascularization, thin skin, bony prominences and small muscle mass are some of the reasons for this fact.

The traditional microsurgical reconstruction algorithm is proposed for most raw areas of the region. The advent of the study of perforator flaps,^1^ ^,^ ^2^ however, brought to the reconstructive surgeon's arsenal new local flaps as the flap propeller.^3^ Taken together, the medical literature has been making contributions on the rapprochement with old patchwork with technical modifications,^4^ allowing transposition of the largest tissue islands, longer range and less potential damage to the donor site. In this topic, Georgescu^5^ added a new concept to the definition of Microsurgery, the microsurgical dissection without actual microvascular anastomosis.

The extensor digitorum brevis muscle (EDB) has been used as interposition tissue in surgical technique for the treatment of tarsal coalition since 1927.^6^ ^,^ ^7^ In these cases it was used more like gliding than as an actual island flap.

EDB flap was first applied in 1973 by Barfred and Reumert^8^ to cover a wound of the lateral malleolus. It has been highlighted in microsurgical way for reconstruction as functional transplantation for chronic facial paralysis,^9^ ^,^ ^10^ after being replaced by the use of the pectoralis minor, serratus anterior and gracilis.

From its description, few reports have been published in the literature^11^ ^-^ ^16^ and only in 2003, Martinet *et al*., ^17^ Chattar-Cora and Pederson^18^ and Chateau *et al*.^19^ published a significant number of cases, with respectively 15, 20 and 52 patients operated on this technique and with good results. From 2009, we started our personal clinical experience with the flap.

The aim of the study was to evaluate retrospectively the results obtained in patients undergoing surgery in which we use EDB as skin muscle flap coverage and as tissue to fill cavities after surgical treatment of chronic osteomyelitis in the foot, ankle and distal leg, as well as to determine its clinical feasibility and analyze possible complications especially on the donor area.

We did not find in the national literature searched (SciELO and LILACS databases) any report of this technique.

## MATERIALS AND METHODS

In the period between November 2009 and July 2012 eleven patients were operated with the EDB flap technique, nine men and two women, aged between 10 and 66 years old. Indications included treatment of wound raw area related to acute trauma in four patients and post-traumatic osteomyelitis in seven patients. The defects were covered with small flaps ranging from 3x3 to 6x3 cm^2^.

**Figure 1:**
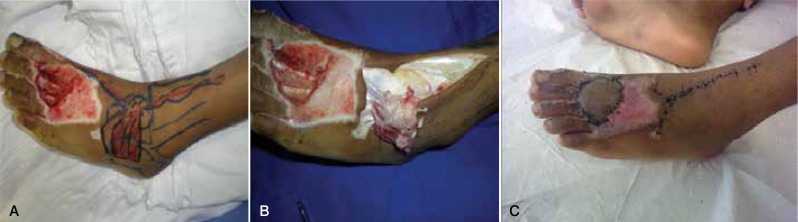
(Patient #2) (A) Skin defect along the 1st and 2nd commissures with drawing of the graft planning, (B) Retail dissected with ligation of the anterior tibial vessel. (C) After skin grafting.

**Figure 2:**
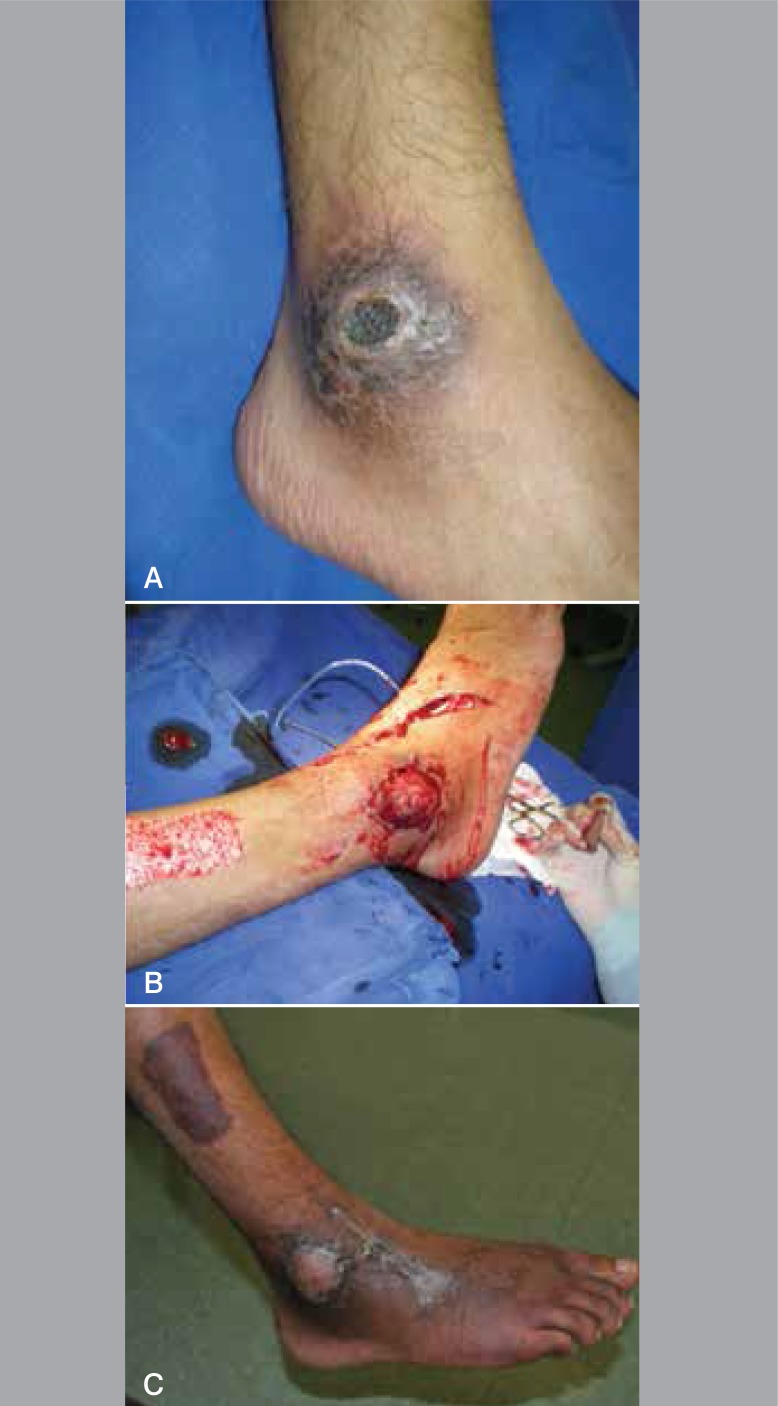
(Patient #4) (A) Skin necrosis on the right lateral malleolus (B) Retail positioned via a subcutaneous tunnel and donor site in healing process (C) Retail and donor area well healed.

In two patients the flap was a reverse flow to cover the forefoot. (Figure 1) In nine patients the flap was anterograde. (Figure 2)

Of the nine anterograde flaps, in five patients the flap was transposed to the medial region of the distal end of the leg. In four patients the flap was applied to the lateral face of the ankle. In one patient (patient # 9) a random fasciocutaneous flap was associated concomitantly.

In both retrograde flaps (i.e., when ligature of the anterior tibial artery is done and the flow comes from the first dorsal metatarsal artery) flaps covered the first commissure and the dorsum of the foot, respectively.

Patients were evaluated according to the following criteria: viability of the flap, resolution of skin defect, healing of chronic infection, dehiscence of the donor area and the need for additional procedures. As subjective criteria, we assessed the satisfaction level of the patient on the procedure.

## RESULTS

All patients operated with the technique included in the study had a minimum follow-up of 12 months, ranging from 1 to 4 years after surgery.

Ten of eleven flaps were viable. There were flaps with partial suffering. The unviable flap was completely lost. In all cases, except for the loss, there was complete healing of the skin defect and cure of chronic osteomyelitis with EDB flap during follow-up, with no recurrence episodes.

We had problems with dehiscence of the donor site in five of 11 patients (45% of patients with viable flaps). Of the five patients with dehiscence of the donor area, four progressed to complete healing only with outpatient dressings (superficial wounds). In a patient who presented with exposure of tendons and bones, it was necessary to use a reverse sural flap to cover the donor site on the dorsum of the foot.

For the patient who presentad total flap loss, a new flap was required (a reverse flow sural chimeric flap, with a portion of the lateral gastrocnemius muscle). After this procedure, there was skin coverage healing, cure of osteomyelitis and bone consolidation.

We believe that EDB flap loss was likely caused by vascular damage related to prior dissection of the anterior compartment of the distal end of the leg by local bone graft.

All patients were satisfied with functional and aesthetic results. The patient that had complete loss of the flap was also satisfied, since he considered that there was minimal damage to the donor site.

The results are summarized in Table 1.

**Table 1:** Characteristics of results from eleven patients operated in this study.

P #	Gender	Age (years)	Etiology	Loss location	Area (cm^2^)	Date of surgery	Complications
1	F	33	osteomyelitis (post-traumatic)	Ankle lateral	6x3	Nov/24/2009	*Piod gangrenosum*
2	M	10	Trauma	1ª commissure foot	3x3	Dec/29/2009	None
3	M	38	osteomyelitis (post-traumatic)	Ankle lateral	3x3	Jan/11/2010	Donor site dehiscence
4	M	24	osteomyelitis (post-traumatic)	Ankle lateral	3x3	Mar/2/2010	None
5	M	57	osteomyelitis (post-traumatic)	1/3 dist leg	3x3	Aug/12/2010	None
6	F	42	osteomyelitis (post-traumatic)	1/3 dist leg	3x3	Jan/12/2011	Donor site dehiscence
7	M	40	osteomyelitis (post-traumatic)	1/3 dist leg	3x3	Feb/4/2011	Total loss
8	M	66	osteomyelitis (post-traumatic)	1/3 dist leg	3x3	Feb/1/2012	Donor site dehiscence
9	M	39	Exposition of synthesis mat.	Ankle lateral	3x3	Jul/3/2012	Donor site dehiscence
10	M	29	osteomyelitis (post-traumatic)	1/3 dist leg	3x3	Jul/13/2012	None
11	M	50	trauma (compound fracture)	Dorsal forefoot	3x3	Jul/25/2012	Donor site dehiscence

P =Patient.

## DISCUSSION

The elevation of the flap based on the anterior tibial artery / 1^st^ dorsal metatarsal allowed for perfect positioning of the muscle along the defects.

Like any muscle flap, dissection plans are quite clear and this facilitates their lifting. Also like any muscle flap it is necessary to remove a skin graft blade, sacrificing additional donor area.

Our results corroborate data from the literature,^17^ ^-^ ^19^ which consider EDB pedicle flap in the anterior tibial artery or the first metatarsal dorsal artery feasible and safe.

In the case of loss (patient #7), it was necessary to dissect the proximal pedicle of the flap in a region previously addressed by surgery, affected by fibrosis and adhesions. The authors consider that this should be avoided at all costs, switching the indication for surgery in these situations.

The rate of problems in the donor area of the muscle was very high. Although most of them (4/5 dehiscence) has been solved only with dressing changes, in the most time consuming of them (patient #11), the complete healing of the donor site occurred at 12 weeks, which definitely delayed full rehabilitation of the patient. The dehiscence and delayed wound healing is probably related to vascular compromise of the flap surface integument of EDB (perforating cutaneous vessels). In the most dramatic case (patient #9), it was necessary to use a reverse sural flap for treatment of donor site. This patient presented with extensive skin necrosis in the region. We associated the magnitude of cutaneous suffering in the donor area to the type of incision to lift the flap of the EDB. The rectilinear longitudinal accesses cause less cutaneous vascular suffering. Moreover, "L" shaped incisions or with some angle or vertex, associated with a higher skin suffering by causing major damage to vessels and the skin septal subdermal vascular system.

**Figure 3:**
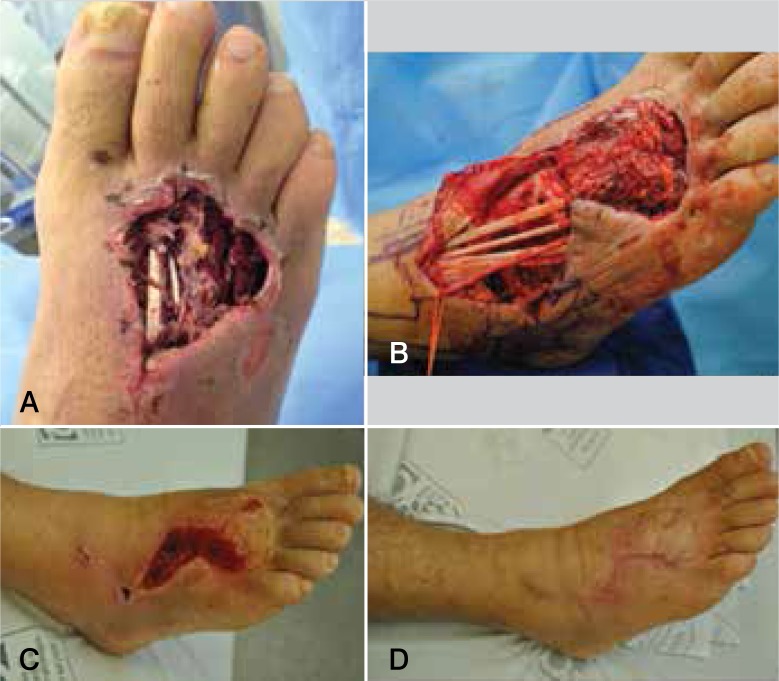
(Patient #11) (A) Postoperative aspect of compound fracture of the metatarsals with bone-tendon exposure and plates after skin necrosis. (B) "L" shaped access, dissection of EDB flap and positioning over the wound. (C) Good integration of blade flap and graft, showing, however, dehiscence of borders in the donor area. (D) Complete wound healing after 12 weeks.

**Figure 4:**
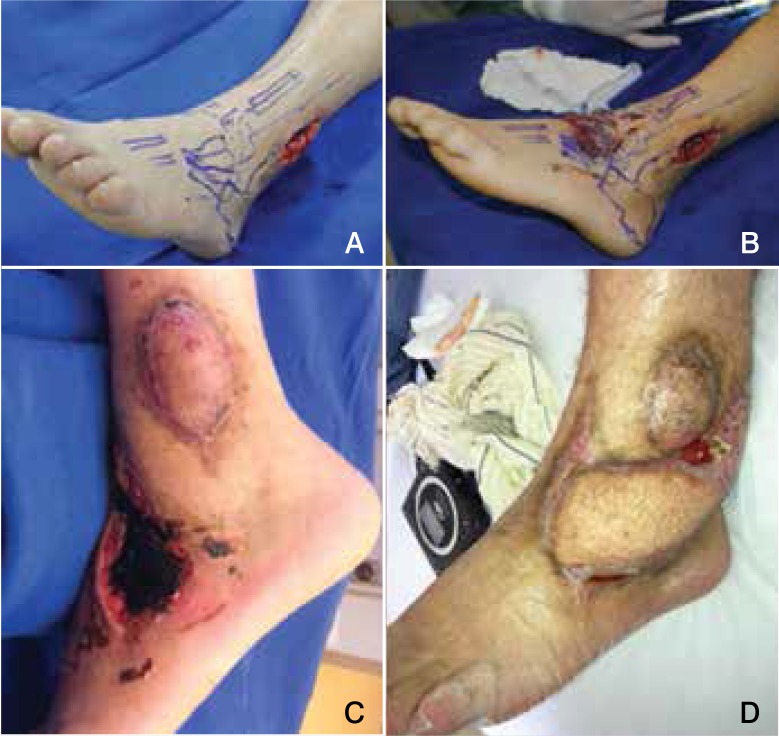
(Patient #9) (A) Planning of the flap and defect with exposure of the plate in the lateral malleolus (B) Dissected graft, it is noted that the incision made an "L" (C) Necrosis of the skin overlying the muscle donor area (D) postoperative of sural flap healed

Although the rectilinear longitudinal incisions may hinder flap elevation, we considered prohibitive "L" shaped incisions on the donor area of the EDB muscle. (Figures 3 and 4) Likewise, we also disagree with Kim *et al.* ^20^ that describes the use of two parallel incisions for flap elevation, as a solution for skin suffering observed in the donor area. We believe that the technique has been described in only three cases, all about the muscle with reverse flow. The examples in this paper do not represent the actual clinical practice found in the majority of cases in the literature. We consider the high risk of skin suffering in the range of parallel incisions due to the involvement of irrigation skin after removal of the muscle.

The authors continue performing EDB flap as a top choice for small defects of the foot and ankle, as well as cavitary osteomyelitis of the distal 1/3 of the leg.

## CONCLUSÃO

The EDB flap is feasible and safe for foot, ankle and distal leg losses. There is the possibility of suffering, dehiscence and delayed healing of the end flap donor area of EDB.

Such complications can be minimized with incisions that preserve the cutaneous vasculature of the donor area (rectilinear longitudinal incisions).
